# Reduced Resting-State Functional Connectivity in Current and Recovered Restrictive Anorexia Nervosa

**DOI:** 10.3389/fpsyt.2017.00030

**Published:** 2017-03-28

**Authors:** Jessica Clare Scaife, Lauren Rose Godier, Nicola Filippini, Catherine J. Harmer, Rebecca J. Park

**Affiliations:** ^1^Department of Psychiatry, University of Oxford and Oxford Health NHS Foundation Trust, Warneford Hospital, Oxford, UK; ^2^FMRIB Centre, University of Oxford, Oxford, UK

**Keywords:** anorexia nervosa, fMRI, resting-state, neural connectivity, gray matter

## Abstract

Functional connectivity studies based on resting-state functional magnetic resonance imaging (rs-fMRI) have shown alterations in brain networks associated with self-referential processing, cognitive control, and somatosensory processing in anorexia nervosa (AN). This study aimed to further investigate the functional connectivity of resting-state networks (RSNs) in homogenous subsamples of individuals with restrictive AN (current and recovered) and the relationship this has with core eating disorder psychopathology. rs-fMRI scans were obtained from 12 female individuals with restrictive AN, 14 females recovered from restrictive AN, and 16 female healthy controls. Independent components analysis revealed a set of functionally relevant RSNs, previously reported in the literature. Dual regression analysis showed decreased temporal coherence within the lateral visual and auditory RSNs in individuals with current AN and those recovered from AN compared to healthy individuals. This decreased connectivity was also found in regions associated with somatosensory processing, and is consistent with reduced interoceptive awareness and body image perception, characteristic of AN. Widespread gray matter (GM) reductions were also found in both the AN groups, and differences in functional connectivity were no longer significant when GM maps were added as a covariate in the dual regression analysis. This raises the possibility that deficits in somatosensory and interoceptive processing observed in AN may be in part underpinned or exacerbated by GM reductions.

## Introduction

Anorexia nervosa (AN) is a psychiatric disorder in which the relentless and compulsive pursuit of self-starvation leads to severe malnourishment. Recovery rates are low and around a quarter of those affected go on to develop a chronic course of the disorder (1). It has the highest mortality rate of any psychiatric disorder (2) and is one of the most debilitating diseases in young women (3). AN remains one of the most challenging psychiatric disorders to treat, particularly in adults (4). There are few evidence-based psychological treatments (5) and very little evidence that there are pharmacological agents which give benefit (6–8). In the quest to develop novel interventions, there has been increasing interest in disease mechanisms (9, 10), in particular, neurobiological factors and processes underlying AN (11, 12). A neurobiological account of AN based on dysfunctional ventral limbic and dorsal executive circuitry has been proposed (11), but relatively few studies have directly investigated aberrancies in the neural circuitry of individuals with current AN.

This study used resting-state functional magnetic resonance imaging (rs-fMRI), in which the temporal synchronicity of spatially separate brain regions is measured. This cross-brain synchronicity is thought to reflect functional networks integral to information processing (13). fMRI blood oxygenation level-dependent (BOLD) signal is measured in individuals at rest, while performing no particular task. BOLD signals are then correlated in time (14). Resting-state networks (RSNs) are comprised of regions which show strong temporal coherence and are thus believed to be functionally connected (15).

There are a number of different approaches to analyzing resting-state data. In the present study, we used a whole-brain independent components analysis (ICA), a data-driven approach which does not require *a priori* regions of interest and looks at the patterns of temporal synchronicity between brain regions which emerge while the brain is at rest (16, 17). Along with a few studies which have also used whole-brain ICA, the AN literature also contains RSN studies using other approaches, such as seed-based analysis, network-based ICA, and graph analysis. Differences in both methodology and population studied have limited direct comparisons between studies [see the review by Gaudio and colleagues (18)].

In the first published study of rs-fMRI in individuals recovered from AN, our group found increased connectivity within the default mode network (DMN) between the precuneus and dorsolateral prefrontal cortex (DLPFC) using whole-brain ICA (19). The DMN incorporates parts of the prefrontal cortex including the DLPFC, the cingulate cortex, and the precuneus and is more active at rest than during active tasks (20). It is believed to be involved in stimulus-independent thought and self-reflection. The DLPFC and anterior cingulate are also involved in cognitive control and may be responsible for excessive control over appetite and food reward in AN (11, 21, 22). Increased connectivity of the insula and the DMN in those with a current diagnosis of anorexia nervosa has also been reported (23), which may normalize in recovery (24). However, some RSN changes do persist into recovery, such as changes in the connectivity of regions in the frontoparietal network (24) and our finding of increased connectivity in the DMN (19).

Differences in networks implicated in cognitive control have also been observed, including increased connectivity of the DLPFC and ventromedial PFC, regions associated with cognitive inflexibility (25). Reduced connectivity of the inferior frontal gyrus (IFG) within the ventral attention network has also been reported (26). The IFG is part of the cognitive control system of the brain which governs response inhibition (27). Furthermore, alterations in connectivity between the thalamus and the prefrontal cortex have also been seen in AN, which may play a role in the cognitive dysfunction seen in this group (28). In a study using whole-brain ICA to examine RSNs in a population of adolescents with AN, decreased functional connectivity between the executive network and the anterior cingulate was reported, which might contribute to impaired cognitive flexibility in relation to the control of appetite and body image in AN (29).

Consistent with the changes in body image perception characteristic of AN, altered connectivity patterns associated with disturbed body image have also been seen. Favaro and colleagues found decreased connectivity in the ventral-visual network in both acute and recovered AN groups and increased connectivity in the somatosensory network in the acute AN group. These results were correlated with visuospatial abilities and were suggested to reflect the inability to integrate visual and somatosensory perceptual information. This inability might sustain body image disturbance in AN (30). Phillipou and colleagues also found decreased functional connectivity between sensorimotor and visual networks in AN (31). A number of studies have also reported changes in the insula in AN: increased functional connectivity between the insula and the cerebellum in the cerebellar–parietal network (32) and reduced connectivity with the thalamus has been reported (33, 34). These findings may help to explain the impairments in body awareness and body perception in AN.

This study is a novel application of the exploratory whole-brain ICA approach in a group which includes a sample of restrictive currently ill individuals and a group who have recovered from AN. Only two previous studies have used whole-brain ICA to study resting-state functional connectivity in AN, and these studies were methodologically different to this study. In our previous study (19), the resting-state scan followed a disorder-related task, which may have impacted on rumination. Furthermore, we previously only included a recovered sample. In the study by Gaudio and colleagues (29), only adolescents with only a short history of AN were included. Functional differences between this group and healthy controls (HCs) are likely to be less extreme than in those who have experienced chronic AN.

In this study, we sought to address a number of limitations in our previous study (19). While our previous approach of studying individuals recovered from AN (21) has the advantage of removing the confounding effect of acute starvation on neural activity (11), including an acutely ill sample in the current study may allow us to begin separating impairments associated with a history of AN from those associated with the starvation state. We also focus exclusively on understanding restrictive AN, reducing heterogeneity of the AN sample. Restrictive and binge/purge subtypes of AN are associated with cognitive and neurobiological differences (35), and by isolating a purely restrictive sample, we aimed to lower within-group variation, thereby allowing between-group differences to be more easily detected. In contrast to our previous study, the resting-state scan for this study was carried out prior to any task components, to avoid the potential effects of task in the activity of RSNs.

In this study, we predicted that with this more homogenous sample and in the absence of any bias from a previous symptom-provocation task, RSNs associated with somatosensory and interoceptive processes will show changes in connectivity, particularly in the current AN group, consistent with altered body image perception and interoception reported in the illness (10).

## Participants and Methods

### Participants

Forty-two participants were recruited for three experimental groups: 12 female individuals with anorexia nervosa (AN; restrictive subtype), 14 recovered (AN-R) females, and 16 female HCs (Table 1). General exclusion criteria for all subjects were left-handedness and magnetic resonance imaging (MRI) contraindications. General inclusion criteria for the HC group were a normal body mass index (BMI) (18.5–25), maintained for the 12 months prior to the study and Eating Disorder Examination Questionnaire (EDE-Q) scores within 1 SD of the global mean scores for young women, no evidence of current or past psychiatric disorder, no history of neurological or significant medical illness, and no first degree relative with a current or past eating disorder diagnosis. Inclusion criteria for the AN-R included a previous DSM-IV diagnosis of AN, absence of significant eating disorder pathology for at least 12 months prior to the study (e.g., restricting, excessive exercise, bingeing, purging), maintenance of a BMI within normal range (18.5–25) for the 12 months prior to the study, and EDE-Q scores within 1 SD of the EDE-Q global mean scores for young women. Inclusion criteria for the AN group included a current DSM-IV (Diagnostic and Statistical Manual of Mental Disorders, fourth Edition) diagnosis of anorexia nervosa, confirmed using the eating disorder examination interview and being currently underweight in BMI (<17.5, calculated during the screening session).

**Table 1:** **Demographics, mood questionnaire and eating disorder scores in the three groups (M ± SD, one-way ANOVA *p*-scores)**.

Sociodemographic data	Healthy Controls (HCs) (*N* = 16)	Recovered (AN-R) (*N* = 14)	Current AN (AN) (*N* = 12)	*p*-Score
Age	24.3 ± 5.7	27 ± 6.5	29.4 ± 6.0	0.092
Body mass index	21.2 ± 2.0	20.9 ± 1.6	15.4 ± 1.9	<0.001*
National Adult Reading Test	109.6 ± 6.8	114.6 ± 6.8	114.7 ± 8.3	0.159
Age of onset of anorexia nervosa (AN) (years)	N/A	16.5 ± 2.1	20.1 ± 5.9	0.04
Duration of AN (years)	N/A	5.8 ± 4.2	10.3 ± 5.2	0.02
EDE	0.2 ± 0.2	0.7 ± 0.6	2.8 ± 1.6	<0.001*
Visual Analog Scales (VAS) “thinness”	47.5 ± 15.3	54.8 ± 11.2	52.0 ± 27.9	0.6
VAS “fear of weight gain”	31.9 ± 19.9	54.9 ± 24.1	79.4 ± 19.9	<0.001**
STAI-State	26.9 ± 6.5	34.4 ± 5.4	51.2 ± 15.5	<0.001*
STAI-Trait	32.9 ± 9.7	45.2 ± 10.6	61.7 ± 14.0	<0.001**
BDI	3.3 ± 4.5	6.4 ± 6.4	30.3 ± 18.7	<0.001*
YBC-EDS- SRQ current	N/A	3.8 ± 3.9	15.2 ± 7.7	<0.001
YBC-EDS-SRQ past	N/A	23.8 ± 5.3	23.3 ± 5.6	0.82

Post hoc differences between AN and HCs indicated by *, between both AN and HC and AN-R and HC indicated by **. Where two groups were compared, p-Score is from independent samples t tests.

*BMI, body mass index; NART, National Adult Reading Test; STAI, State-Trait Anxiety Inventory; BDI, Beck Depression Inventory; EDE, eating disorder examination; VAS, Visual Analog Scales; YBC-EDS-SRQ, Yale–Brown–Cornell Eating Disorder Scale Self-Report Questionnaire*.

In the AN group, primary diagnosis was AN; however, there were some comorbidities: two also fulfilled criteria for comorbid Major Depressive Disorder (MDD), one currently suffered from comorbid Generalized Anxiety Disorder (GAD), and five presented with both MDD and GAD. In the AN group, five participants were on SSRI/SNRI antidepressant drugs, and three participants were also taking atypical antipsychotic drugs. In the AN-R group, three participants were taking SSRI/SNRI antidepressant drugs.

### Measures

The Structured Clinical Interview for DSM-V Axis I Disorders (36) was used to screen for DSM-V Axis-I disorders. ED psychopathology was measured using the Eating Disorder Examination (37), which refers to the previous 28 days. During screening, participants completed the Yale–Brown–Cornell Eating Disorder Scale Self-Report Questionnaire (YBC-EDS-SRQ) (38), which indexes eating-related preoccupations and or rituals.

Depressive symptoms were measured using the BDI-II (39). Anxiety symptoms were measured using the State-Trait Anxiety Inventory (40) on the day of screening, which is a 40-item self-report instrument that captures both state and trait anxiety. Verbal IQ was measured using the National Adult Reading Test (NART) (41), with non-native English speakers excluded from group means.

On the day of the scan, participants were asked not to eat for 4 hrs prior to the fMRI scan and to drink only water or calorie-free drinks. Participants completed Visual Analog Scales for the dimensions “how do you see yourself” (very thin–very fat) and “fear of weight gain” (0–100 mm, not at all–extremely) to assess feelings about body image immediately prior to the scan.

### Neuroimaging Protocol

Scanning was performed at the University of Oxford, Centre for Clinical Magnetic Resonance Research (OCMR) using a 3-T Siemens Trio scanner with a 32-channel head-coil. The neuroimaging protocol comprised functional and structural sequences as follows.

### Resting-State Functional Magnetic Resonance Imaging

Whole-brain functional imaging was performed using a gradient-echo EPI sequence (TR = 2,000 ms, TE = 28 ms, flip angle = 89°, field of view = 224 mm, voxel dimension = 3 mm × 3 mm × 3 mm, acquisition time = 6 min 4 s). For the resting-state scan, subjects were instructed to lie in dimmed light with their eyes open, think of nothing in particular, and not to fall asleep.

A functional scan involving a food-pictures task was performed following the resting-state scan, prior to the structural MRI, data are not reported in this paper, see Ref. (42).

### Structural MRI

Structural MRI was acquired at the end of the two functional MRI sequences. 3D high-resolution T1-weighted MR images were acquired using a magnetization-prepared rapid gradient-echo sequence (TR = 2,040 ms, TE = 4.7 ms, flip angle = 8°, field of view = 192 mm, voxel dimension = 1 mm isotropic, acquisition time = 6 min).

### Analysis of RSNs

Resting-state functional magnetic resonance imaging analysis of resting-state data was carried out using Multivariate Exploratory Linear Optimized Decomposition into Independent Components (MELODIC, part of FSL: http://www.fmrib.ox.ac.uk/fslmelodic/) (43). Individual pre-processing consisted of motion correction, brain extraction, spatial smoothing using a Gaussian kernel of full width at half maximum (FWHM) 5 mm and high pass temporal filtering with a cut-off of 150 s (0.007 Hz). rs-fMRI volumes were registered to the individual’s structural scan and standard space images using Oxford Centre for Functional MRI of the Brain’s (FMRIB) Non-linear Image Registration Tool (FNIRT). This data analysis was performed in three stages:

First, the pre-processed functional data containing 180 time-points for each subject were temporally concatenated across subjects in order to create a single 4D dataset. Then, the (group-wise) concatenated multiple rs-fMRI datasets were decomposed using a group ICA to identify large-scale patterns of functional connectivity in the population of subjects.

Second, dual regression was carried out on the data, which allowed voxel-wise comparisons of resting functional connectivity to be made between subjects (44). Within each subject’s rs-fMRI dataset, subject-specific temporal dynamics and spatial maps that are associated with each group ICA map were identified. This is a two-step process, which involves first the use of the full set of group-ICA spatial maps in a linear model fit (spatial regression) against the separate rs-fMRI datasets, resulting in matrices describing temporal dynamics for each component for each subject. Second, these subject-specific time-course matrices are used to perform temporal regression (linear model fit) against the associated rs-fMRI dataset to estimate subject-specific spatial maps. In the final step of the group ICA, the different component maps are collected across subjects into single 4D files (one per original ICA map, with the fourth dimension being subject ID). At this stage, independent components of interest were identified, and subjected to further analysis.

Third, a voxel-wise general linear model (GLM) based analysis was used to assess group differences in each spatial map using permutation-based non-parametric testing (5,000 permutations) (45) with cluster-based thresholding using Threshold-Free Cluster Enhancement (TFCE) and a family-wise-error corrected cluster significance threshold of *p* < 0.05 applied to the suprathreshold clusters. This results in spatial maps characterizing the between-subject/group differences. The GLM comparison included the groups of interest comparison (current AN vs controls, recovered AN vs controls, current and recovered AN vs controls). Each of the contrasts of interest was tested for group averages and difference between groups.

Non-parametric tests were used to safeguard against the possibility that the between-subjects effects were non-Gaussian, and because such non-parametric inference has greater robustness against spatial non-stationarity than commonly used parametric methods (46). Because previous evidence suggested that acquisition of rs-fMRI data after a task may potentially confound results (47), we acquired our data prior to the food image task, which is not reported in this paper.

### Analysis of Structural MRI

Whole-brain analysis was carried out using a voxel-based morphometry-style analysis (FSL-VBM) (48) using default settings as described at www.fmrib.ox.ac.uk/fsl/fslvbm/. In brief, brain extraction and tissue-type segmentation were performed and resulting gray matter (GM) partial volume images were aligned to standard space using first linear (FLIRT) and then non-linear (FNIRT) registration tools. The resulting images were averaged, modulated, and smoothed with an isotropic Gaussian kernel of 5 mm FWHM to create a study-specific template, and the GM images were re-registered to this, including modulation by the warp field Jacobian. In the figures, the result maps are reported in accordance with neurological convention (right is right).

Voxel-wise GLM analysis was carried out using permutation testing in a similar fashion as described for the rs-fMRI analysis. Permutation-based non-parametric testing (5,000 permutations) (45) with cluster-based thresholding using TFCE and a family-wise error (FWE)-corrected cluster significance threshold of *p* < 0.05 applied to the suprathreshold clusters.

For each subject, GM, white matter (WM), and cerebrospinal fluid (CSF) volumes were derived and computed as a percentage of total brain volume. This was compared between groups and GM volume in the AN group was correlated with BMI, age, lowest BMI, duration of disease and past YBC-EDS-SRQ scores.

Statistical analyses of non-imaging variables were carried out using SPSS software (SPSS, Inc., Chicago IL, USA) version 22.0. Threshold for statistical significance was set to *p* < 0.05.

### Covariates

Structural images were used as additional covariates on a voxel by-voxel basis to interrogate rs-fMRI data. GM images of each subject were extracted using FMRIB’s Automated Segmentation Tool (http://fsl.fmrib.ox.ac.uk/fsl/fslwiki/FAST/), registered to standard space, smoothed to match the intrinsic smoothness of the rs-fMRI data, voxel-wise demeaned across all subjects in both groups together and added as a confounding regressor (nuisance) to the GLM design matrix used to analyze rs-fMRI data. Adding GM maps reduces variance in the data due to potentially confounding anatomical differences between subjects (49). Due to the (non-significant) trend toward group differences in age (*p* = 0.092), a subsequent analysis also included age as a covariate. The results did not survive the inclusion of age as a covariate. When examining group differences in SPSS, age was added as a *post hoc* covariate.

## Results

### Demographic and Psychological Characteristics

Table 1 shows the demographic and psychological characteristics of the groups. Duration of illness was significantly greater in the AN than the AN-R group: *t*(1,24) = −2.45, *p* = 0.02. Age of onset was significantly later in the AN than the AN-R group: *t*(1,24) = −2.17, *p* = 0.04. Lowest BMI, past YBC-EDS-SRQ scores, and NART did not differ between groups (*p* > 0.05). The AN group reported significantly higher state anxiety and depression (BDI) levels than HC group. The AN group also reported higher trait anxiety than the AN-R group. The AN-R group also had higher trait anxiety than HC but state anxiety and depression were not significantly different. There was a significant group effect on the measure “how afraid are you of gaining weight”. Both AN groups were significantly more afraid of gaining weight than HC (*F* = 17.0, *p* < 0.001), the AN group were also more afraid of gaining weight than the AN-R group (*p* < 0.05).

### Resting-State Functional Connectivity

**Figure 1:**
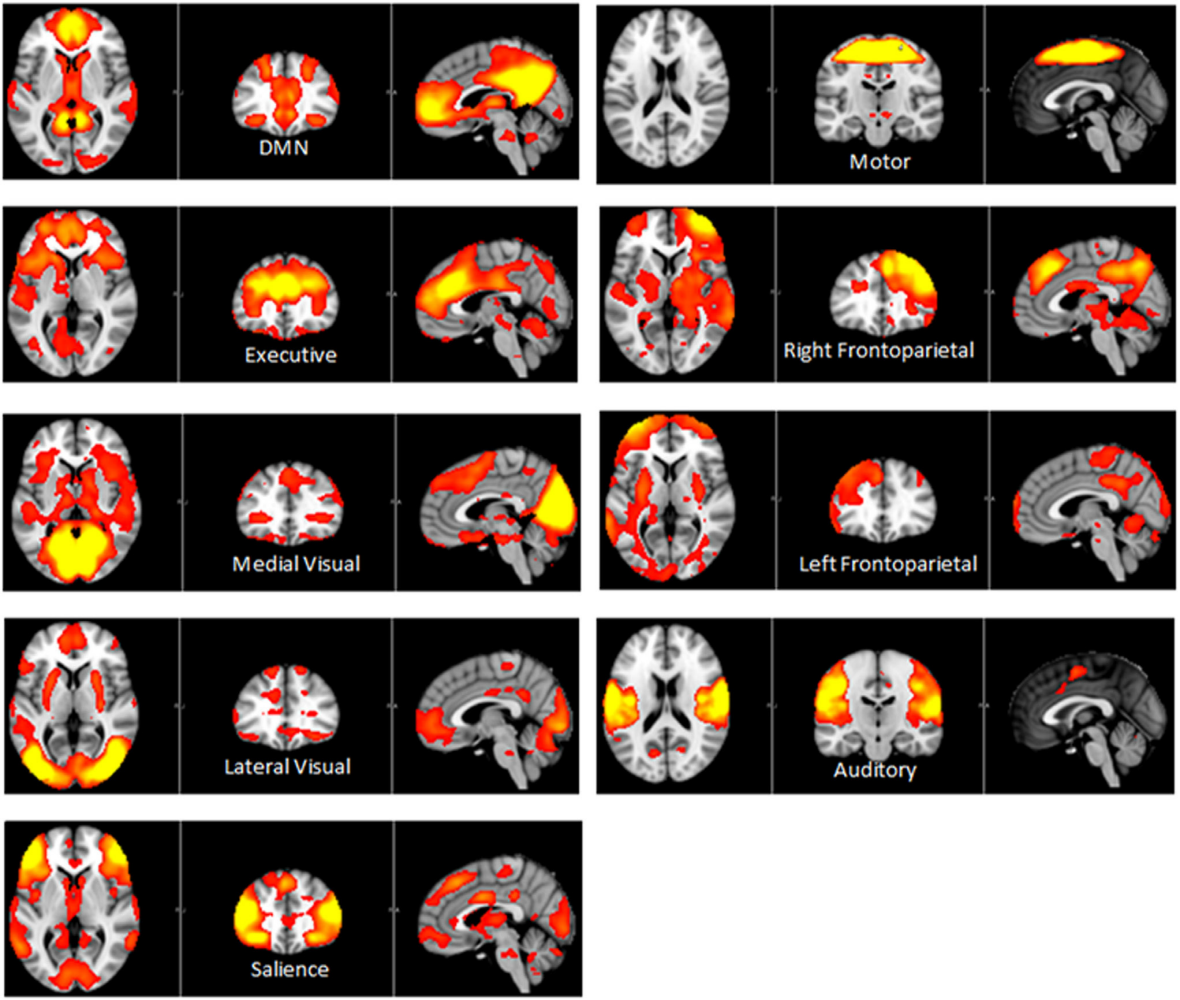
**Axial, coronal and sagittal slices for the main RSNs detected, overlaid onto the standard MNI brain**.

Independent component analysis (ICA) defined 33 independent components. Of these, nine components were identified as RSNs (covering the majority of GM) and were evaluated further (Figure 1, shows nine RSNs). The other components reflected distinct artifacts resulting from head motion and physiological or scanner noise. The RSNs of interest included: DMN, salience, medial visual, lateral visual, auditory, motor, executive control, and frontoparietal (right and left). These networks corresponded to RSNs described previously with high stability over time (43).

**Figure 2:**
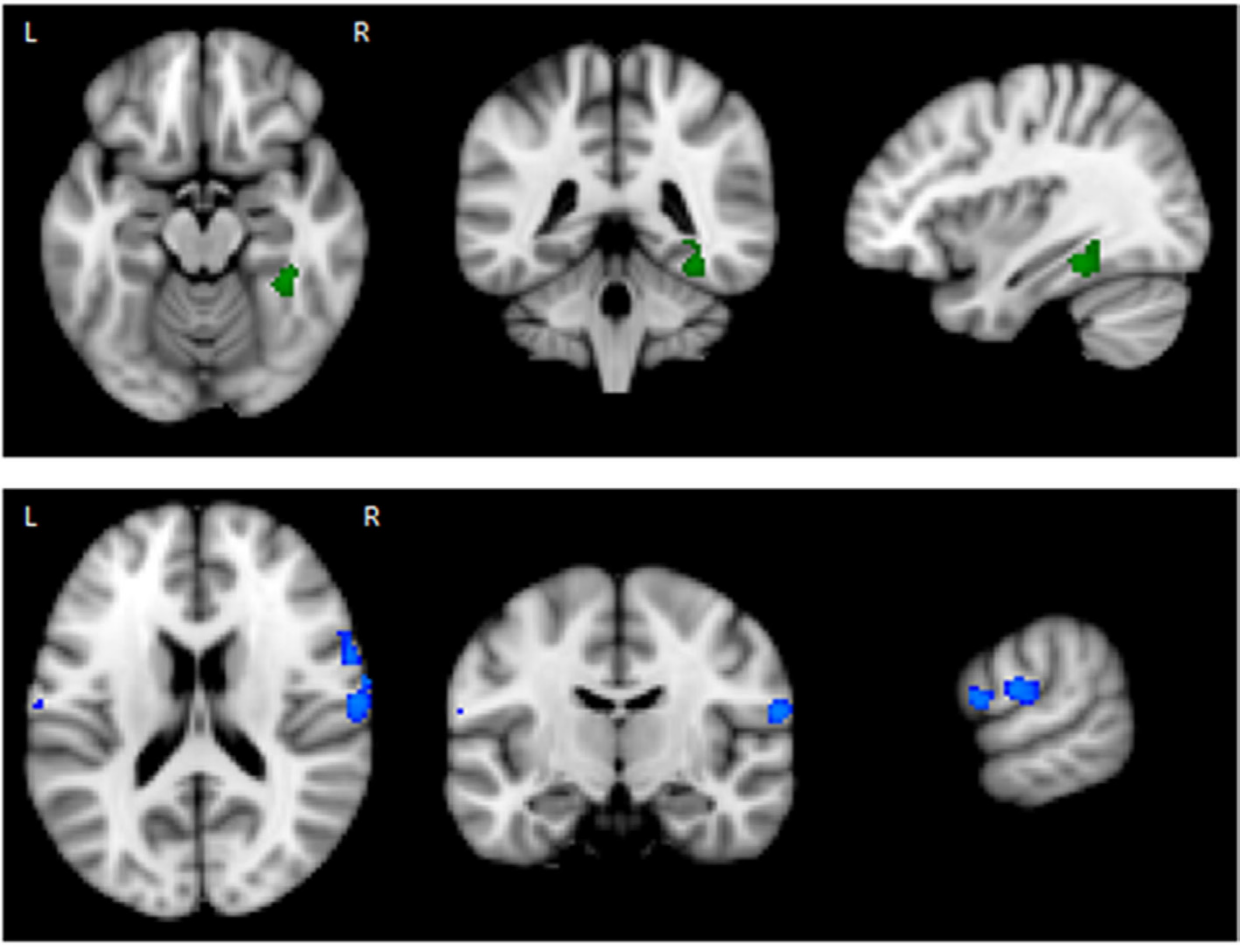
**Top: Regions of decreased connectivity with the Lateral Visual Network, the temporal fusiform cortex is shown. Bottom: Regions of decreased connectivity within the Auditory Network, the precentral and postcentral gyri are shown**.

**Figure 3:**
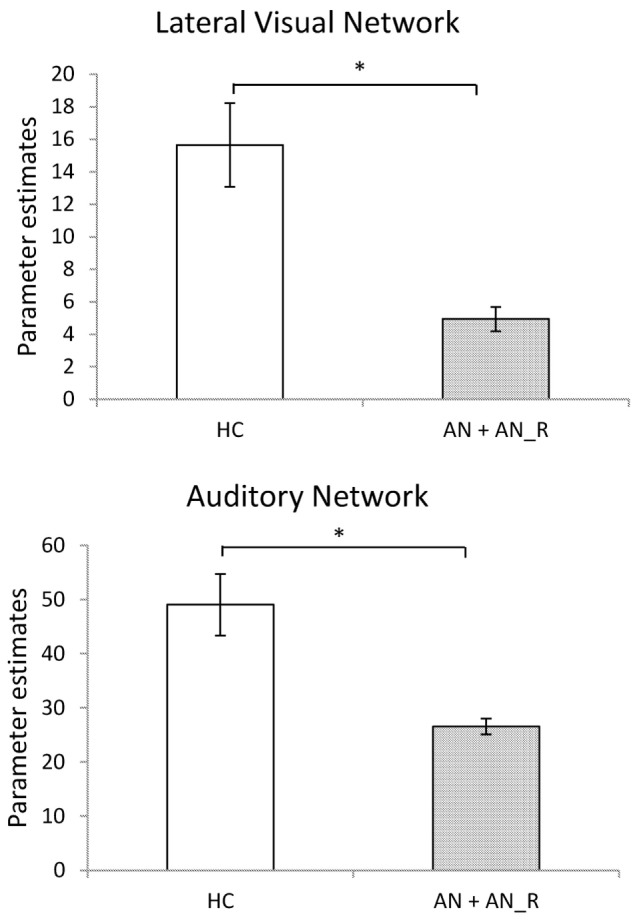
**Significantly reduced functional connectivity in the pooled AN and AN-R groups compared to the HC group in the Lateral Visual Network (Top) and Auditory Network (Bottom)**.

**Figure 4:**
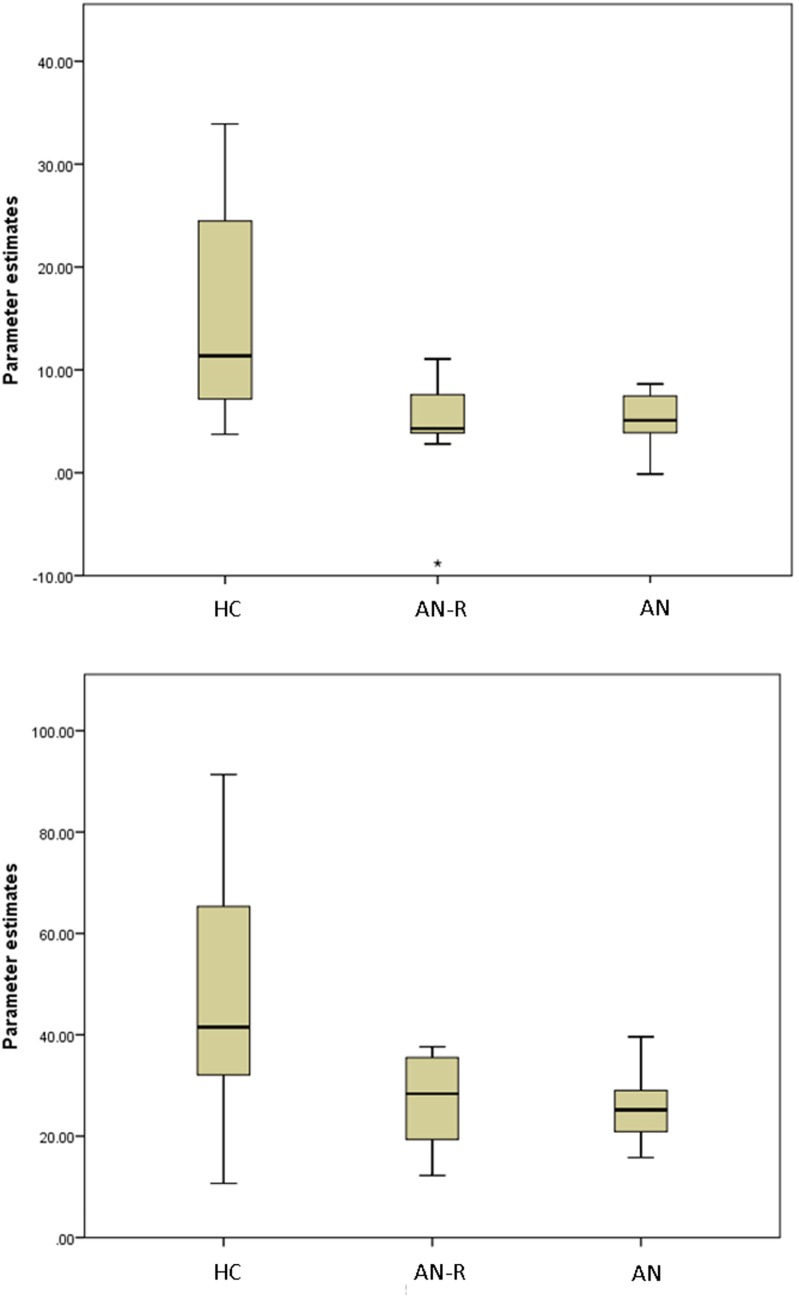
**Box plots of extracted BETA values in the HC, AN-R and AN groups in the Lateral Visual Network (Top) and Auditory Network (Bottom)**. Asterisk represents an outlier in the group.

The results of the GLM found significant between-group differences in the voxel-wise spatial distribution of the functional connectivity maps in the lateral visual and auditory networks. Significantly decreased temporal correlation (coherence) was observed in the contrast: HC greater than AN and AN-R within the auditory network in clusters in the following regions: the right insula extending into the central opercular cortex, the left opercular cortex, right Heschl’s gyrus, the right precentral gyrus extending into the inferior frontal and postcentral gyri, the left precentral and postcentral gyri (peak 64, −12, 18, *t* = 4.44, voxel size 819; see Figures 2 and 3). Significantly decreased temporal correlation (coherence) was observed in AN and AN-R groups compared to HC group within the lateral visual network in the temporal fusiform cortex extending into the temporal–occipital fusiform cortex and lingual gyrus (peak 38, −36, −14, *t* = 4.04, voxel size 176; see Figures 2 and 3). A box plot of the extracted values for all three groups, is shown in Figure 4.

Total scores on the YBC-EDS-SRQ (past) did not significantly correlate with either RSN dual regression output *p* > 0.05.

### Structural MRI

The data for global CSF, WM, and GM volumes are reported in Table 2. There was no significant between-group difference in WM volume (*F*[2,39] = 0.8, *p* = 0.45). There was a significant between-group difference in CSF volume (*F*[2,39] = 6.83, *p* = 0.004), driven by an increase in CSF volume in the AN group compared with AN-R (*p* = 0.032) and HC (*p* = 0.004).

**Table 2:** **Global gray matter (GM), white matter (WM) and cerebrospinal fluid (CSF) volumes expressed as a percentage of total brain volume in the three groups (M ± SD, one-way ANOVA *p*-scores)**.

	HC *N*(16)	AN-R *N*(14)	AN *N*(12)	*p*-Score
WM volume	37.5 ± 1.4	37.8 ± 1.1	37.1 ± 1.3	0.45
GM volume	43.0 ± 1.1	42.2 ± 1.6	40.0 ± 4.5	0.013
CSF	19.5 ± 1.6	20.0 ± 2.2	22.2 ± 2.3	0.004

**Figure 5:**
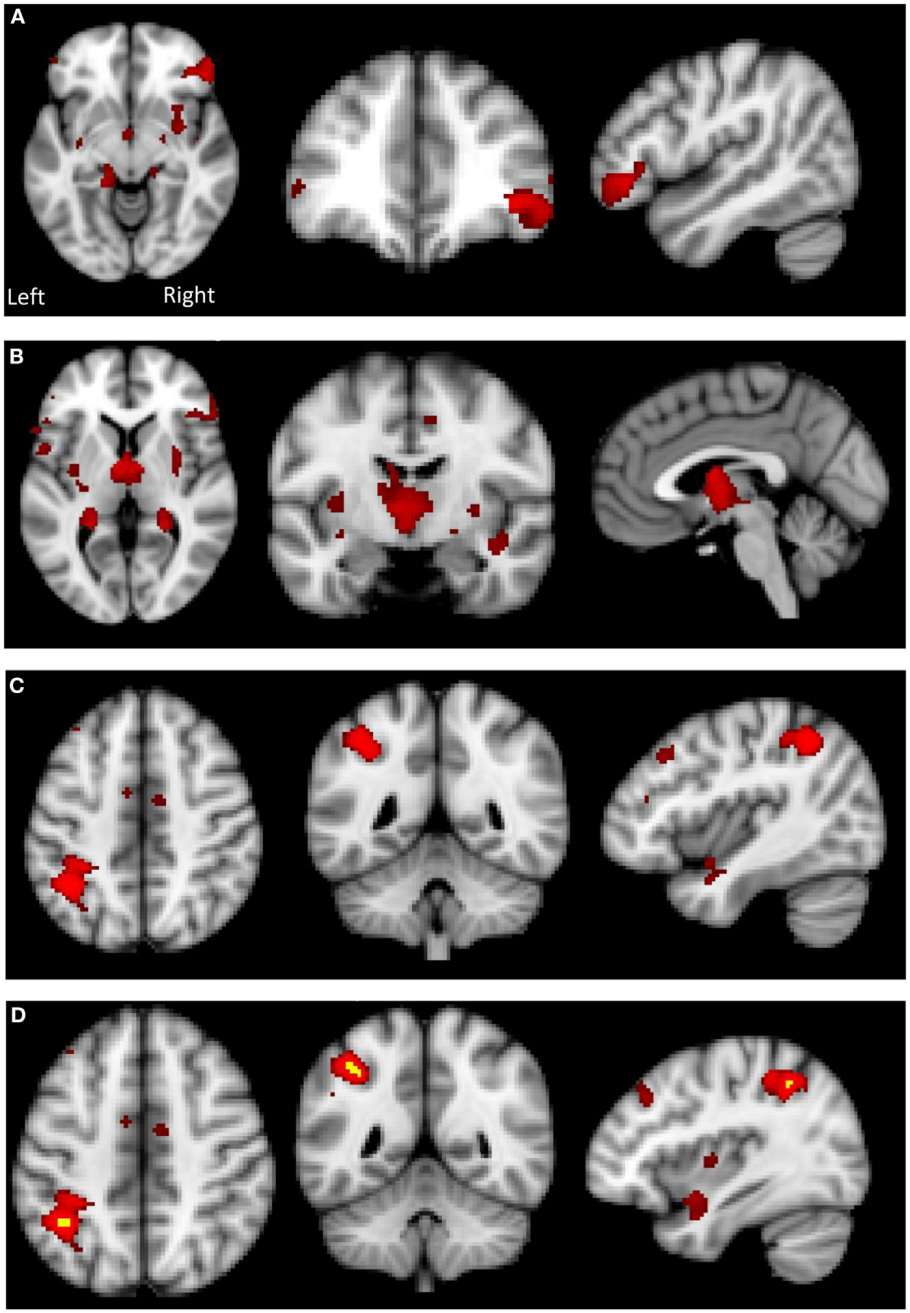
**Top: gray matter (GM) reduction in the anorexia nervosa (AN) group compared to HC in (A) the lateral prefrontal cortex, (B) putamen and thalamus, (C) left supramarginal gyrus/superior parietal lobule. Bottom: (D) overlap in GM reduction in the AN group (red) and the AN-R group (yellow) in the left supramarginal gyrus/superior parietal lobule**.

There was a significant between-group difference in GM volume (*F*[2,39] = 4.868, *p* = 0.013) driven by reduction in GM in the AN group compared with HC (*p* = 0.012). There was no significant difference in global GM between the AN group and the AN-R group (*p* = 0.093). In the results of the GLM, the contrast HC > AN revealed a widespread reduction in GM in the brains of individuals with AN, with significant clusters in the left precentral gyrus, left middle frontal gyrus, bilateral inferior frontal gyrus, right lateral prefrontal cortex, dorsal anterior cingulate/juxtapostional lobule, left planum polare-insular cortex, bilateral thalamus, bilateral hippocampus, bilateral putamen, left angular gyrus/supramarginal gyrus, and superior parietal lobule *p* < 0.05 (Figure 5). There were no brain regions in which the AN group had increased GM density volume compared with AN-R and HC groups.

**Figure 6:**
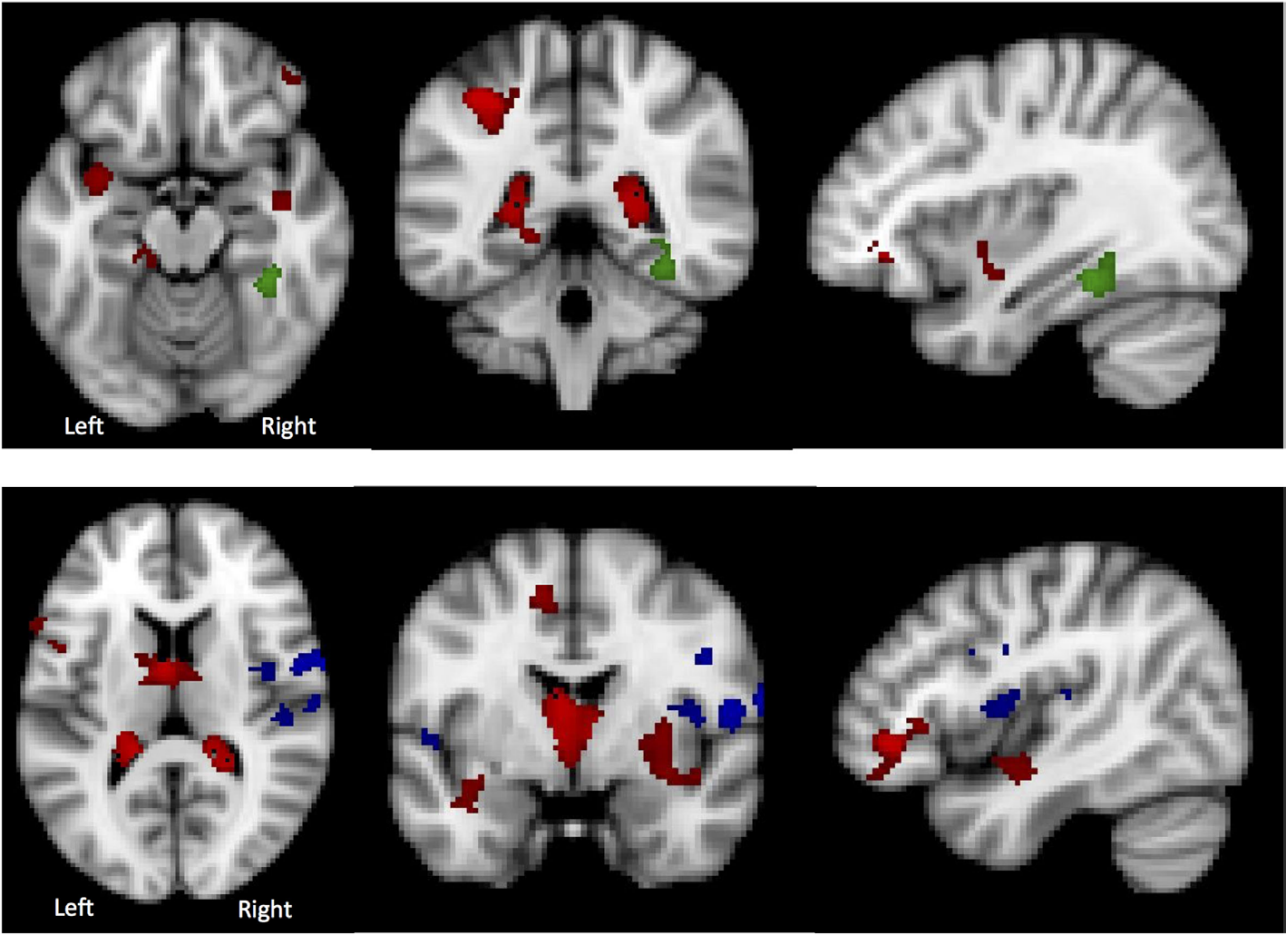
**Top: in the Lateral Visual Network (Green), the region in which there is a group difference is distinct from those regions in which there are significant gray matter (GM) differences (HC > AN) (shown in red). Bottom: in the Auditory Network (blue), the region in which there is a group difference is distinct from those regions in which there are significant GM differences (HC > AN) (shown in red)**.

Global GM volume did not significantly differ between the AN-R and HC groups; however, in the GLM (contrast HC > AN-R), there was a significant cluster in the right supramarginal gyrus/superior parietal lobule, *p* < 0.05 (Figure 5). The overlap in regions of GM reduction in AN and AN-R groups is seen in Figure 6.

### GM Maps as Covariates

To determine whether BOLD differences at rest between the groups were influenced by the significant GM reduction in the AN and AN-R groups, GM maps were added as covariates (nuisance variables) in the RSN fMRI analysis model (GLM). The BOLD-related group differences in the lateral visual and auditory networks (reported above) did not survive correction for GM. One possible explanation for this is that GM differences account for a significant part of the group differences in functional RSN data. Another possibility is that as adding GM as a covariate changes the degrees of freedom in the model, such that the results no longer reach significance. No significant correlations were found between the percentage signal change in the functional data and the GM values for the same areas. There was also virtually no overlap between those regions found to be significant in the RSN analysis and those significantly with reduced GM (Figure 5). This suggests that the group differences in RSN coherence are genuine and not the result of GM differences.

## Discussion

The results of this study suggest that regions associated with interoceptive and somatosensory functioning, including the processing of body image information, show hypoconnectivity in individuals with a history of AN (both current and past). As the recovered AN group were not currently malnourished, the observed differences cannot be solely explained by starvation-associated brain atrophy. Rather, these results suggest that AN is associated with enduring functional differences in brain organization, which could be either a scar of the illness or a premorbid feature, which represents an illness-related biomarker. In contrast to our previous resting-state study in recovered AN (19), we did not observe any differences in the DMN network. This could be due to methodological differences, as we previously measured resting-state neural activity after a symptom-provocation paradigm, which may have primed disorder-related preoccupation and ruminations associated with DMN activity.

The lateral visual network was described by Beckman and colleagues (43) as one which includes lateral visual cortical areas: the occipital pole, occipito-temporal junction, and superior parietal regions. This network has been implicated visual or visuo-spatial attention (50). We found, decreased connectivity in this network within the right temporal/temporal–occipital fusiform cortex, an area associated with face and body recognition (51). The fusiform gyrus is shown to activate in response to body images in both healthy individuals and in EDs (52, 53); however, a decreased response in this region has been found in those with AN (52). Decreased connectivity between the fusiform body area (the posterior region of the fusiform gyrus) and extrastriate body has previously been reported in AN and correlated with body image distortion (54). Consistent with our findings, Favaro and colleagues also reported decreased connectivity in both acute and recovered AN within the visual network, in a region also associated with visual perception of the body (30). In a study using a network-based statistical approach, Ehrlich and colleagues found decreased connectivity in a thalamo-insular subnetwork including the fusiform gyrus, which might explain the disturbance in homeostatis and perceived body state in AN (55). Our results, together with previous literature, suggest that brain circuits associated with body image processing are dysfunctional in AN, and may underpin features such as distorted body image perception and abnormal experience of the body, characteristic of AN.

The auditory network includes the primary and secondary auditory cortices, insular cortex, the anterior cingulate cortex, anterior supramarginal gyrus, and thalamus (43). There have been few papers which have examined the role of auditory resting state network and these have focused on its role in tinnitus (56, 57). There have, to date been none reporting any changes in its connectivity in AN. In this study, within the auditory network, decreased connectivity was found in a number of regions including the precentral and postcentral gyri. The precentral and postcentral gyri correspond to the primary motor and somatosensory regions, respectively, and interact to contribute to complex motor and somatosensory functions (58, 59). Dysfunction of both these regions have been reported in AN in response to viewing other women’s bodies (60), and increased activity within the precentral gyrus is seen in AN in response to viewing happy facial expressions (61). In addition, reduced connectivity of the postcentral gyrus with the sensorimotor network has previously been reported in AN compared to HC, suggested to reflect reduced somatosensory responsiveness in AN (62). In the McFadden study, reduction of postcentral gyrus activation in AN-R group compared to HC failed to reach significance, but a reduction in the postcentral gyrus in the AN group compared to the AN-R group was found—a difference which we did not see.

Decreased connectivity of the insula within the auditory network was also observed. Dysfunction of the insula has often been reported in neuroimaging studies of AN (63–66). The insula is a region of the brain with multiple complex functions, and is extensively connected to many regions in the brain, making it central to neural communication (67, 68). There is consistent evidence of interoceptive deficits and insula dysfunction in AN (12). The insula is implicated in the regulation of appetite (69), processing of taste information (70), and sensitivity to pain (71), all of which are shown to be aberrant in AN (72–74). Interestingly, in the context of the other regions of altered functional connectivity reported in this study, the insula has been associated with interoceptive processing, and providing a sense of the physiological condition of the body (75), the dysfunction of which could underpin distorted body image perception in AN (68).

We performed a VBM on the structural data, a technique which allows investigation of local concentrations of brain tissue, through voxel-wise comparison of multiple brain images. This approach requires no *a priori* hypotheses about regions of possible differences between groups, and it allows comprehensive measurements throughout the entire brain.

We found reduced GM in parts of the postcentral gyrus and insula cortex in the AN group. Reductions in these regions were no longer seen in the AN-R group, while a reduction in the supramarginal/angular gyrus region was shown in both AN groups. This raises the possibility that although there may be structural recovery from atrophy caused by malnourishment in regions responsible for interoceptive awareness of the bodily state and body image perception, functional changes may have taken place, which do not recover. Indeed, deficits in interoceptive processes and body image perception are also found in individuals recovered from AN (76, 77). In line with our results, investigations of GM differences in AN have often reported widespread reductions. A recent systematic review reported reductions in the insula, frontal operculum, occipital, medial temporal, and cingulate cortex, but emphasized the inconsistency of results across samples and methodologies (78). In line with our finding of global GM loss in the AN but not the AN-R group, brain volume has also been shown to increase following weight gain and recovery (79–81), further supporting the possible role of starvation-related brain atrophy in the psychopathology of AN.

This study has several methodological limitations of note, as well as some important strengths. While our participants were asked to fast overnight prior to the resting-state scan to control for levels of hunger, ED research has been criticized for failing to control for the effects of dehydration and starvation, particularly in relation to brain matter volume (82). The effects of this were shown in a study by Frank and colleagues (83), in which GM volume was investigated in a sample of individuals with AN who had been on a strict meal plan for 1–2 weeks, in order to avoid the effects of malnutrition. Controlling for age, comorbid diagnoses, and brain volume, the authors found increased volume in the orbitofrontal cortex and right insula in AN (current and recovered) compared to controls (83). These results emphasize the importance of controlling for malnutrition and global brain volume in future AN research.

As with many studies in this area, the sample size used was fairly small. However, we did isolate a sample of purely restrictive AN. A number of resting-state studies in AN have included both restrictive and binge/purge AN subtypes and, as such, are unable to control for the possibility of within-group variability in brain network activity, which may be related to important differences in psychopathology between subtypes of AN. However, future research may benefit from larger sample sizes enabling the inclusion and comparison of both AN subtypes. In addition, a number of participants had comorbid anxiety or depression diagnoses and, as such, were taking serotonergic antidepressants and/or antipsychotic medications. Antidepressant and antipsychotic medications have been shown to alter resting neural connectivity (84–86), and so the effects of these medications, or indeed comorbid diagnoses, on our results cannot be ruled out.

## Conclusion

Consistent with our predictions and with clinical characteristics of AN we have demonstrated decreased connectivity in regions associated with somatosensory and interoceptive function, as well as body image processing in individuals with current AN and those recovered from AN. These results are consistent with clinical and research findings of deficits in interoceptive awareness in AN, abnormalities in the way the body is experienced, and body image distortion. Despite normal global GM volumes in recovered AN individuals, whose weight is fully restored, functional changes in RSNs remain. These may contribute to more persistent body image issues and potentially represent a vulnerability to relapse. Future research would benefit from larger sample sizes allowing the comparison of AN subtypes, and stricter controls on nutrition and hydration prior to imaging.

## Ethics Statement

This study was carried out in accordance with the recommendations of NRES South Central—Oxford A Research Ethics Committee. The protocol was approved by the NRES South Central—Oxford A Research Ethics Committee.

## Author Contributions

JS, LG, CH, and RP designed research; JS, LG, and NF acquired and analyzed the data; JS, LG, CH, NF, and RP wrote the manuscript.

## Conflict of Interest Statement

CH has received consultancy fees from Lundbeck, P1vital, Astra Zeneca and Servier. She is a company director of Oxford Psychologists Ltd. and holds shares in the same company. CH has received grant funding from UCB, J&J, Astra Zeneca, Lundbeck, and Sunovion. JS, LG, NF, and RP report no biomedical financial interests or potential conflicts of interest.
